# Tezepelumab attenuates exertional symptom burden in severe asthma: insights from a real-world cohort

**DOI:** 10.3389/falgy.2026.1849268

**Published:** 2026-06-29

**Authors:** Slagjana Stoshikj, Katharina Marth, Daniela Boryshchuk, Robin Ristl, Andreas Renner, Christina Bal, Karin Patocka, Wolfgang Pohl, Karin Vonbank, Marco Idzko

**Affiliations:** 1 Clinical Department of Pulmonology, Department of Internal Medicine II, Medical University of Vienna, Vienna, Austria; 2 Comprehensive Center for Chest Diseases (CCCD) - Medical University of Vienna, Vienna, Austria; 3 Department of Pulmonology, Karl Landsteiner Institute for Experimental and Clinical Pneumology, Hietzing Hospital, Vienna, Austria; 4 Institute of Medical Statistics, Center for Medical Data Science, Medical University of Vienna, Vienna, Austria

**Keywords:** biologics, exertional symptoms, patient reported outcomes, severe asthma, tezepelumab

## Abstract

**Background:**

Exertional symptom burden is an important yet insufficiently characterised component of morbidity in severe asthma. Tezepelumab, a monoclonal antibody targeting thymic stromal lymphopoietin (TSLP), reduces exacerbations and improves asthma control across phenotypes, but its effects on patient-reported exertional symptoms and physical activity limitation remain unclear.

**Methods:**

We conducted a retrospective study in 24 adults with severe asthma initiating tezepelumab per GINA criteria. The 6-item Asthma Control Questionnaire (ACQ-6), Asthma Control Test (ACT), and mini-Asthma Quality of Life Questionnaire (miniAQLQ) were administered at baseline and at 3 (*n* = 24), 6 (*n* = 20), and 12 months (*n* = 16). Pre-specified exercise-related items were analysed alongside total scores using the Wilcoxon signed-rank test.

**Results:**

At baseline, patients had poorly controlled asthma (median ACQ-6 3.50/6, ACT 11/25, miniAQLQ 3.53/6) and moderate-to-severe airflow limitation (median FEV₁ 57% predicted). Total scores improved progressively over 12 months, with changes at months 6 and 12 exceeding the respective minimal clinically important differences. Exertion-related items showed consistent improvements at least as pronounced as total score changes. The largest improvements were in strenuous activity limitation (miniAQLQ Q12: median change +2.0, *p* = 0.006) and overall activity limitation (ACQ-6 Q3: median change −2.5, *p* = 0.008).

**Conclusion:**

In severe asthma, Tezepelumab is associated with progressive attenuation of exertional symptoms over 12 months of real-world treatment. These associative findings are consistent with mechanistic data and support further prospective studies.

## 1 Introduction

Asthma is a chronic inflammatory airway disease characterized by variable expiratory airflow, airway hyperresponsiveness, and recurrent respiratory symptoms. Severe asthma is a subset of difficult-to-treat asthma that remains uncontrolled despite maximally optimized therapy and management of all modifiable factors, or that worsens when high-dose treatment is tapered (1, 2). Breathlessness is a disabling and often under-recognized symptom in asthma (3), with exertional dyspnoea – including chest tightness and wheeze provoked by physical activity – representing a particularly prevalent yet insufficiently characterised dimension of this burden. Over half of people with severe asthma (53%) experience physically limiting breathlessness despite optimal pharmacotherapy, compared with 31% of those with mild-to-moderate disease (4). Exertional dyspnoea in asthma reflects the combined effects of bronchoconstriction, mechanical constraints from dynamic lung hyperinflation, increased ventilatory effort during exercise, and modulating psychological factors. Notably, resting lung function measures such as FEV₁, together with outcomes from direct bronchial challenge tests, are poor predictors of exertional symptoms and exercise-related physiological changes in asthma (5). In this context, exercise-induced bronchoconstriction (EIB) represents a classic, measurable expression of exercise-related airway hyperresponsiveness, provoked by hyperpnoea-induced airway cooling and drying, with hyperosmolar stress leading to mast cell activation and bronchoconstrictor mediator release (6).

In patients with severe asthma, exertional symptoms extend beyond classical EIB: chronic airway inflammation, mucus plugging, structural remodelling and peripheral airway dysfunction promote expiratory flow limitation and gas trapping, thereby fostering dynamic hyperinflation and heightened ventilatory effort, so that even moderate physical activity readily provokes dyspnoea, chest tightness and wheeze (7–9). Biologic therapies have transformed the management and prognosis of severe asthma (1, 10). While these agents consistently improve asthma control and health-related quality of life, exertional dyspnoea and exercise capacity have not been comprehensively characterized (11).

Consistent with this concept, Lombardi et al. showed that in patients with severe eosinophilic asthma treated with mepolizumab or conventional therapy, 6-minute walk distance was significantly and positively correlated with asthma control and asthma-related quality of life, as measured by the Asthma Control Test (ACT) and Asthma Quality of Life Questionnaire (AQLQ), respectively (12).

Data on biologics and exercise outcomes in severe asthma are limited. A small CPET study in severe allergic asthma treated with omalizumab showed significant improvements in objectively measured exercise capacity, accompanied by gains in health-related quality of life, and confirmed the feasibility and safety of CPET in this population (13).

The clinical relevance of exertional limitation is also reflected in an ongoing phase 4 randomized trial evaluating the effect of dupilumab on exercise capacity in moderate-to-severe asthma (NCT04203797), although results are not yet available. Among currently available biologics, tezepelumab, a human monoclonal antibody targeting thymic stromal lymphopoietin (TSLP) is particularly attractive in this context because TSLP is an upstream epithelial alarmin implicated in airway hyperresponsiveness, mucus plugging and small-airway dysfunction, and tezepelumab has demonstrated broad efficacy across severe asthma phenotypes with improvements in lung function, asthma control and health-related quality of life (1, 14–17). Mechanistic data from studies such as CASCADE have shown that tezepelumab reduces airway eosinophilia, inflammatory cell counts, and airway hyperresponsiveness, positioning it as a modulator of airway biology beyond its exacerbation-preventive effects (15).

These characteristics make tezepelumab a plausible candidate to affect exertional symptom burden, yet its impact on patient-reported exertional dyspnoea and everyday exercise tolerance has not been specifically examined, with only isolated case-based evidence suggesting a potential benefit on exercise-related outcomes (18).

Against this background, we aimed to investigate changes in patient-reported exertional symptom burden in patients with severe asthma before and after initiation of tezepelumab in routine clinical practice.

Specifically, we hypothesised that upstream TSLP blockade may attenuate the propensity of the asthmatic airway to convert physical exertional stress into amplified type 2 inflammatory responses, thereby reducing the burden of exertional symptoms — a dimension of disease impact not systematically characterised under alarmin-targeted therapy to date (19, 20).

## 2 Materials and methods

This observational longitudinal study included adult patients with severe asthma who initiated tezepelumab in routine clinical practice, with severe asthma defined and treatment started according to current GINA criteria. Eligible patients had a confirmed diagnosis of severe asthma and at least one post-baseline visit after ≥ 3 months of therapy. Baseline assessments comprised demographic and clinical characteristics (age, sex, body mass index, asthma duration, comorbidities and maintenance asthma treatment).

Patient-reported outcome measures (PROMs) were obtained using the 6-item Asthma Control Questionnaire (ACQ-6) (21), the Asthma Control Test (ACT) (22) and the mini-Asthma Quality of Life Questionnaire (miniAQLQ) (23).

All instruments were administered at baseline, prior to the first tezepelumab dose, and at routine follow-up visits after 3, 6 and 12 months. In addition to total scores, we identified exercise-related items as variables of primary interest: ACQ-6 items 3 and 6 (activity limitation and rescue bronchodilator use), ACT items 1 and 4 (impact on daily activities and rescue inhaler use), and miniAQLQ items 12–15 (limitations during strenuous, moderate, social and work-related activities), capturing activity limitation and exertional symptoms (Supplementary Table S1). Asthma control categories were defined as ACT ≥ 20 (well controlled), 16–19 (partly controlled), and ≤ 15 (poorly controlled), and as ACQ-6 < 0.75, 0.75–1.5, and > 1.5, respectively. Higher miniAQLQ scores indicate better asthma–specific quality of life. The study was conducted in accordance with the Declaration of Helsinki and applicable local regulatory requirements (EC Number 1075/2024).

### 2.1 Statistical analysis

Descriptive tables were created to summarise patients’ demographic and baseline characteristics. The primary outcomes were PROMs (ACQ-6, ACT and miniAQLQ), which were assessed at baseline and after 3, 6 and 12 months. These scores were summarized using the median and the quartiles. Continuous variables were summarized using the mean and standard deviation for approximately symmetric variables commonly reported on the mean scale, and the median with quartiles for ordinal, bounded or skewed variables, considering the limited sample size rather than relying on formal normality testing. Categorical variables were presented as frequencies and percentages. The distribution of PROMs (ACQ-6, ACT and miniAQLQ) over time was visualised using boxplots. Changes in total scores and individual questionnaire items from BSL to each follow-up time point (months 3, 6 and 12) were assessed using the Wilcoxon signed-rank test for paired samples. Median differences (month - BSL) together with quartiles were reported. Given the exploratory nature of the study, no adjustment for multiple testing was performed. A two-sided *p*-value < 0.05 was considered statistically significant.

## 3 Results

**Figure 1:**
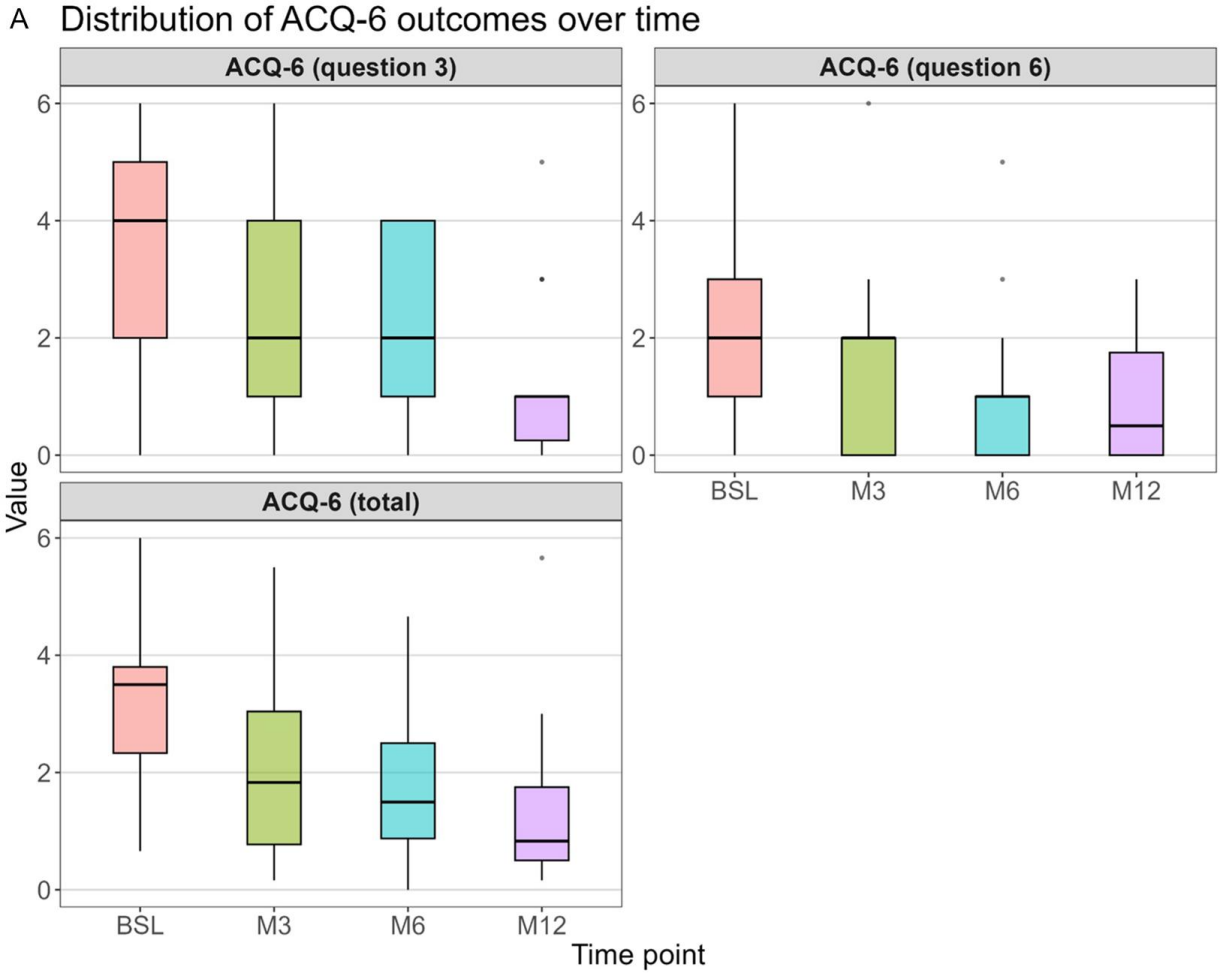

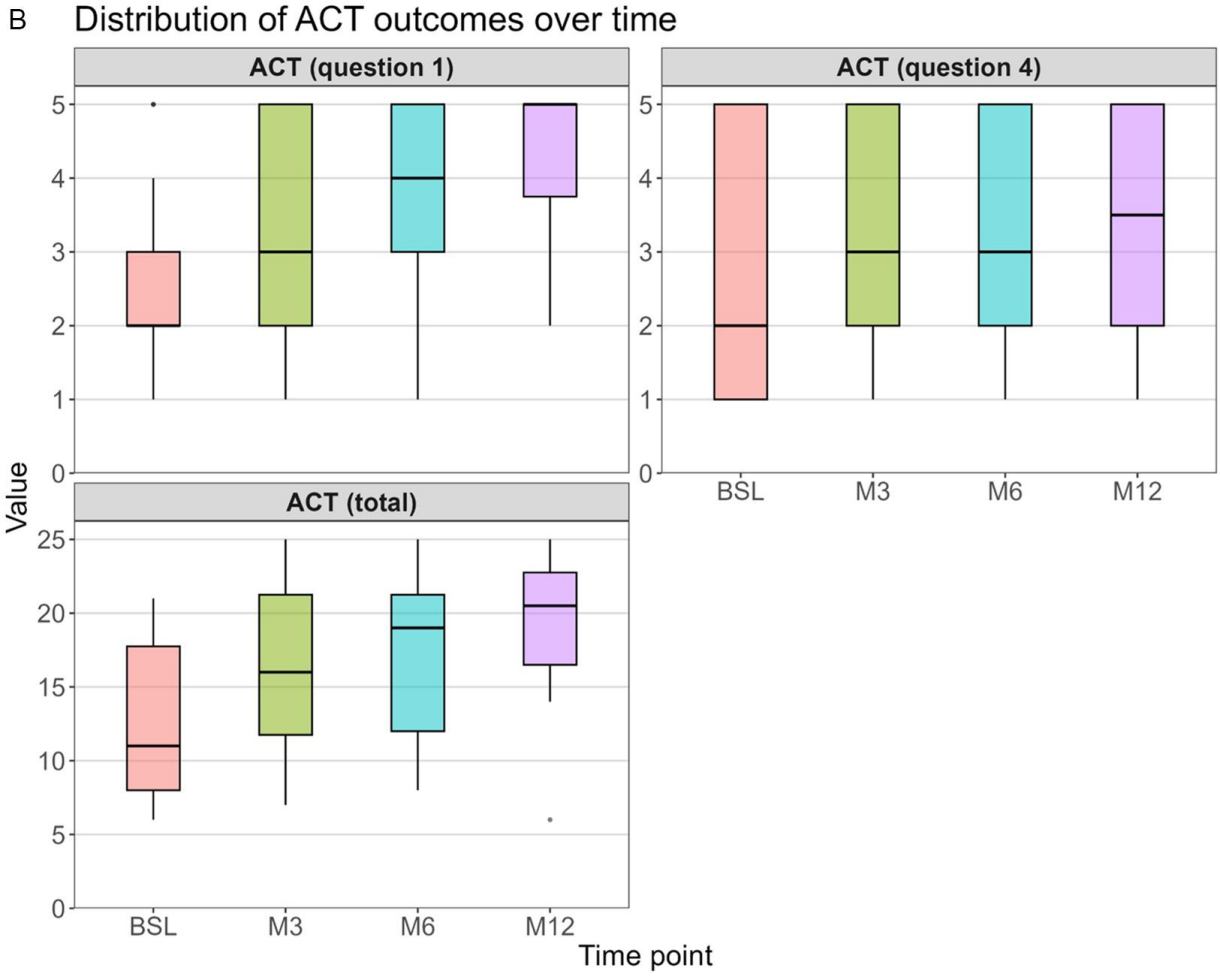

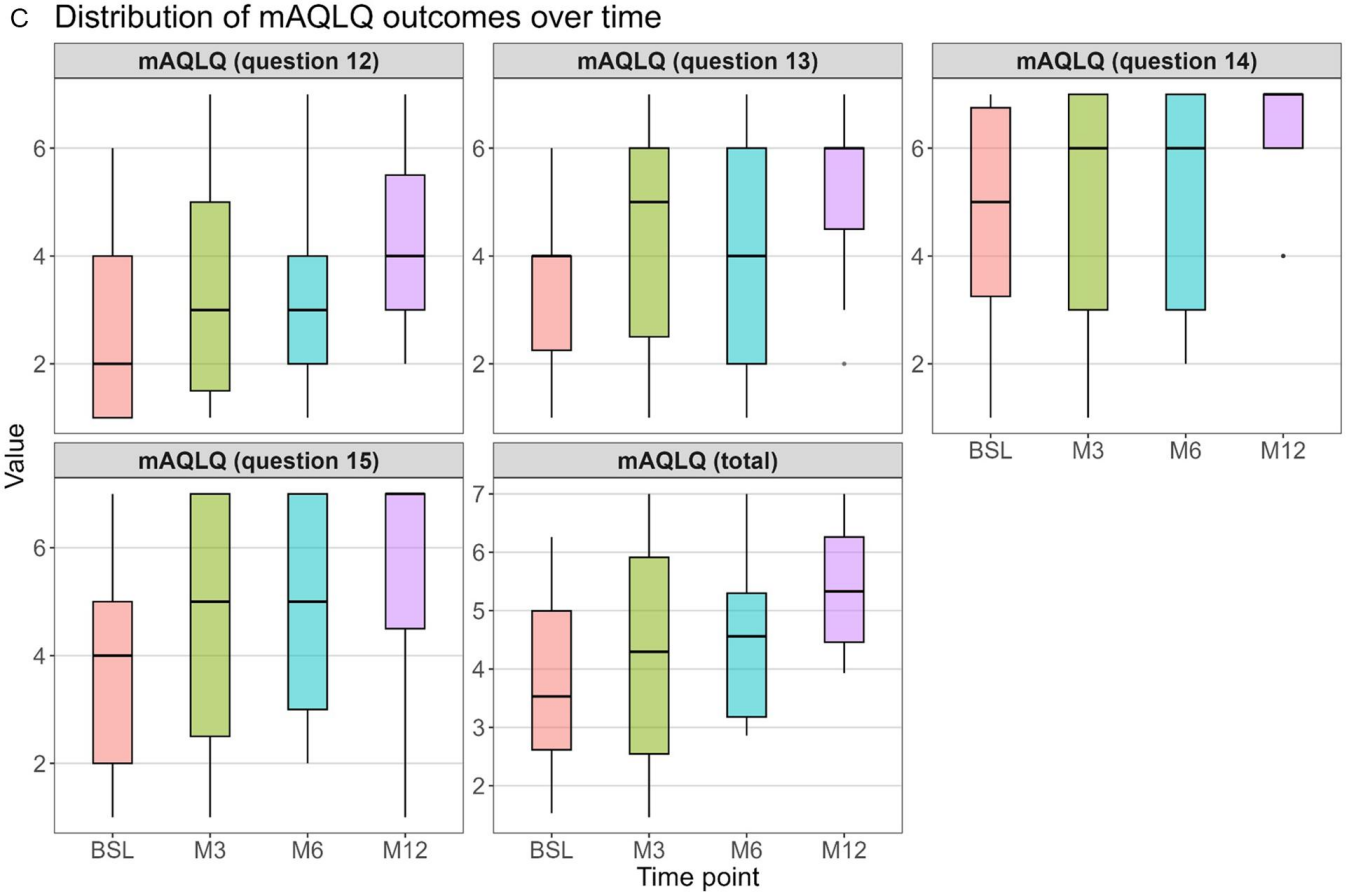
Changes in asthma-related patient-reported outcomes during 12 months of tezepelumab treatment. Panels **(A–C)** show the distribution of total scores for the ACQ-6 **(A)**, ACT **(B)** and miniAQLQ **(C)** at baseline and after 3, 6 and 12 months of tezepelumab treatment in adults with severe asthma in routine care. Scores are displayed as boxplots (median, interquartile range, and range). Lower ACQ-6 scores and higher ACT and miniAQLQ scores indicate better asthma control and asthma-specific quality of life. The horizontal dashed lines indicate the minimal clinically important difference (MCID) for each instrument (ACQ-6 0.5, ACT 3, miniAQLQ 0.5) and, for the ACT, the cut-off for well-controlled asthma (score ≥ 20). Abbreviations: ACQ-6, 6-item Asthma Control Questionnaire; ACT, asthma control test; miniAQLQ, mini-Asthma Quality of Life Questionnaire; MCID, minimal clinically important difference; BSL, baseline; M3, month 3; M6, month 6; M12, month 12.

Twenty-four patients with severe asthma who initiated tezepelumab in routine clinical practice were included. Baseline characteristics are presented in Table 1. The cohort had a mean age of 55.3 ± 15.5 years, and 50% were female. Median asthma duration was 27.5 years (IQR 13.0–44.0), and median FEV₁ was 57.0% predicted (IQR 43.5–76.5), indicating a predominantly moderate-to-severe airflow limitation at baseline. Median blood eosinophil count was 200 cells/µL (IQR 0–300) and median FeNO was 26.0 ppb (IQR 15.0–82.0), reflecting a mixed type 2 inflammatory phenotype. Most patients were on inhaled corticosteroids/long-acting beta-agonists (ICS/LABA) (95.8%), with 58.3% also receiving a long-acting muscarinic antagonist (LAMA); 16.7% required maintenance oral corticosteroids. More than half (54.2%) had received at least one prior biologic therapy. At baseline, median ACQ-6 total score was 3.50 (IQR 2.33–3.80), median ACT total score was 11.0 (IQR 8.0–18.0), and median miniAQLQ total score was 3.53 (IQR 2.60–5.06), confirming poorly controlled asthma with substantially impaired quality of life across all three instruments. All 24 patients completed the 3-month assessment. Data at 6 and 12 months were available for 20 and 16 patients, respectively, primarily reflecting staggered enrolment and ongoing data collection rather than attrition; only one patient was lost to follow-up after month 3, one after month 6, and two discontinued tezepelumab therapy after month 6. Total scores for all three patient-reported outcome instruments improved progressively and significantly over the 12-month follow-up (Tables 2–4, Figure 1). ACQ-6 total score declined from a median of 3.50 at baseline to 1.83 at month 3 (median change −1.0, *p* = 0.003), 1.50 at month 6 (median change −1.34, *p* = 0.001), and 0.83 at month 12 (median change −1.83, *p* < 0.001). ACT total score increased from a median of 11.0 at baseline to 16.0 at month 3 (median change +3.5, *p* = 0.003), 19.0 at month 6 (median change +4.0, *p* < 0.001), and 20.5 at month 12 (median change +7.0, *p* = 0.003), with the 12-month median score exceeding the threshold for well-controlled asthma (≥ 20). miniAQLQ total score improved from 3.53 at baseline to 4.30 at month 3 (median change +0.40, *p* = 0.097), 4.56 at month 6 (median change +0.60, *p* = 0.005), and 5.33 at month 12 (median change +1.30, *p* = 0.007). All changes at months 6 and 12 exceeded the respective minimal clinically important differences (MCID: ACQ-6 of at least 0.5 points (24), ACT ≥ 3 (25), miniAQLQ ≥ 0.5 (23). Exercise-related items showed consistent and clinically meaningful improvements across all three instruments, with changes at 12 months being at least as pronounced as those in total scores (Table 4). For the ACQ-6, item Q3 (activity limitation due to asthma) declined from a median of 4.0 at baseline to 2.0 at month 3 (median change −1.0, *p* = 0.036), 2.0 at month 6 (median change −2.0, *p* = 0.005), and 1.0 at month 12 (median change −2.5, *p* = 0.008). Item Q6 (shortness of breath) declined from 2.0 at baseline, with significant improvements at all three timepoints (month 3: median change −1.0, *p* = 0.009; month 6: median change −1.0, *p* = 0.002; month 12: median change −1.0, *p* = 0.008). For the ACT, item Q1 (ability to get things done at work, school or at home) improved significantly at all timepoints (month 3: median change +0.5, *p* = 0.015; month 6: median change +1.5, *p* = 0.005; month 12: median change +2.0, *p* = 0.024). Item Q4 (rescue inhaler use) showed significant improvement from month 6 onwards (month 6: median change +1.0, *p* = 0.008; month 12: median change +1.0, *p* = 0.020), with no significant change at month 3 (*p* = 0.174).

**Table 1:** Baseline characteristics-overall cohort.

Characteristic	Overall cohort (*n* = 24)
Demographics	
Age, years (mean ± SD)	55.3 ± 15.5
Female sex (*n*, %)	12 (50.0)
BMI, kg/m^2^ (mean ± SD)	26.8 ± 5.1
Smoking status (*n*, %)	
Current	3 (12.5)
Former	10 (41.7)
Never	11 (45.8)
Asthma characteristics	
Age at asthma diagnosis, years (mean ± SD)	26.5 ± 21.5
Asthma duration, years (median [IQR])	27.5 (13.0–44.0)
Exacerbations in previous 12 months, *n* (median [IQR])	2.0 (1.0–3.5)
Hospitalizations for asthma in previous 12 months (*n*, %)	6 (25.0)
Lung function and biomarkers (median [IQR])	
FEV₁, L	1.82 (1.31–2.31)
FEV₁, % predicted	57.0 (43.5–76.5)
Blood eosinophils, cells/µL	200 (1–300)
FeNO, ppb	26.0 (15.0–82.0)
Total IgE, IU/mL	115 (51.3–335.5)
Comorbidities (*n*, %)	
Rhinitis	12 (50.0)
Chronic rhinosinusitis	10 (41.7)
Gastro-esophageal reflux disease	15 (62.5)
Maintenance medications (*n*, %)	
ICS/LABA	23 (95.8)
ICS/LABA/LAMA	14 (58.3)
Maintenance oral corticosteroids	4 (16.7)
Previous biologic therapy (*n*, %)	
Any previous biologic	13 (54.2)
Omalizumab	7 (29.2)
Anti-IL-5/5R	11 (45.8)
Dupilumab	5 (20.8)
Patient-reported outcomes at baseline (median [IQR])	
ACQ-6 total score	3.50 (2.33–3.80)
ACT total score	11.0 (8.0–18.0)
miniAQLQ total score	3.53 (2.60–5.06)

ACQ-6, 6-item asthma control questionnaire; ACT, asthma control test; BMI, body mass index; FeNO, fractional exhaled nitric oxide; FEV₁, forced expiratory volume in 1 second; ICS, inhaled corticosteroids; IgE, immunoglobulin E; IQR, interquartile range; LABA, long-acting beta₂-agonist; LAMA, long-acting muscarinic antagonist; miniAQLQ, mini-Asthma Quality of Life Questionnaire; OCS, oral corticosteroids; SD, standard deviation.

**Table 2:** Exercise-related questionnaire items and total scores at baseline and during tezepelumab treatment.

Questionnaire/Item	Baseline	3 months	6 months	12 months
Scores — median (IQR)				
ACQ-6 Q3: In general, during the past week, how limited were you in your activities because of your asthma?	4.0 (2.0–5.0)	2.0 (1.0–4.0)	2.0 (1.0–4.0)	1.0 (0–1.0)
ACQ-6 Q6: On average, during the past week, how many puffs of short-acting bronchodilator have you used each day?	2.0 (1.0–3.0)	2.0 (0–2.0)	1.0 (0–1.0)	0.5 (0–2.0)
ACT Q1: In the past 4 weeks, how much of the time did your asthma keep you from getting as much done at work, school or at home?	2.0 (2.0–3.0)	3.0 (2.0–5.0)	4.0 (3.0–5.0)	5.0 (3.5–5.0)
ACT Q4: During the past 4 weeks, how often have you used your rescue inhaler or nebulizer medication (such as albuterol)?	2.0 (1.0–5.0)	3.0 (2.0–5.0)	3.0 (2.0–5.0)	3.5 (2.0–5.0)
miniAQLQ Q12: How limited have you been during the past 2 weeks doing strenuous activities (such as hurrying, exercising, running upstairs, sports) because of your asthma?	2.0 (1.0–4.0)	3.0 (1.0–5.0)	3.0 (2.0–4.0)	4.0 (3.0–6.0)
miniAQLQ Q13: How limited have you been during the past 2 weeks doing moderate activities (such as walking, housework, gardening, shopping, climbing stairs) because of your asthma?	4.0 (2.0–4.0)	5.0 (2.0–6.0)	4.0 (2.0–6.0)	6.0 (4.0–6.0)
miniAQLQ Q14: How limited have you been during the past 2 weeks doing social activities (such as talking, playing with pets/children, visiting friends/relatives) because of your asthma?	5.0 (3.0–7.0)	6.0 (3.0–7.0)	6.0 (3.0–7.0)	7.0 (6.0–7.0)
miniAQLQ Q15: How limited have you been during the past 2 weeks doing work-related activities (tasks you have to do at work) because of your asthma?	4.0 (2.0–5.0)	5.0 (2.0–7.0)	5.0 (3.0–7.0)	7.0 (4.0–7.0)
ACQ-6 Total score (MCID ≤0.5)	3.50 (2.33–3.80)	1.83 (0.72–3.08)	1.50 (0.75–2.50)	0.83 (0.50–2.00)
ACT Total score (MCID ≥3, ≥20 = controlled)	11.0 (8.0–18.0)	16.0 (11.5–21.5)	19.0 (12.0–21.5)	20.5 (16.0–23.0)
miniAQLQ Total score (MCID ≥0.5)	3.53 (2.60–5.06)	4.30 (2.46–5.93)	4.56 (3.13–5.33)	(4.46–6.26)

Values are median (IQR). *Δ* denotes change from baseline (follow-up minus baseline). *Δ* 3M/6M/12M = change at 3, 6, 12 months.

MCID sources: ACQ-6 (11), ACT (12), miniAQLQ (13).

ACQ-6, 6-item asthma control questionnaire; ACT, Asthma control test; miniAQLQ, mini-Asthma Quality of Life Questionnaire; BSL, baseline; IQR, interquartile range; MCID, minimal clinically important difference.

**Table 3:** Change from baseline in exercise-related questionnaire items and total scores during tezepelumab treatment.

Change from baseline — median (IQR) with *p*-value (Wilcoxon signed-rank test)				
Questionnaire/Item	Δ 3 months	Δ 6 months	Δ 12 months	Scoring
ACQ-6 Q3: In general, during the past week, how limited were you in your activities because of your asthma?	−1.0 (−2.0 to 0) *p* = 0.036*	−2.0 (−3.0 to 0) *p* = 0.005**	−2.5 (−3.0 to −0.5) *p* = 0.008**	0–6 lower = better
ACQ-6 Q6: On average, during the past week, how many puffs of short-acting bronchodilator have you used each day?	−1.0 (−1.0 to 0) *p* = 0.009**	−1.0 (−2.5 to −1.0) *p* = 0.002**	−1.0 (−2.0 to −0.5) *p* = 0.008**	0–6 lower = better
ACT Q1: In the past 4 weeks, how much of the time did your asthma keep you from getting as much done at work, school or at home?	+0.5 (0 to 1.0) *p* = 0.015*	+1.5 (0 to 2.0) *p* = 0.005**	+2.0 (0 to 3.0) *p* = 0.024*	1–5 higher = better
ACT Q4: During the past 4 weeks, how often have you used your rescue inhaler or nebulizer medication (such as albuterol)?	0 (0 to 1.0) *p* = 0.174	+1.0 (0 to 2.0) *p* = 0.008**	+1.0 (0 to 2.0) *p* = 0.020*	1–5 higher = better
miniAQLQ Q12: How limited have you been during the past 2 weeks doing strenuous activities (such as hurrying, exercising, running upstairs, sports) because of your asthma?	0 (0 to 2.0) *p* = 0.026*	0 (−1.0 to 3.0) *p* = ns	+2.0 (0 to 3.0) *p* = 0.006**	1–7 higher = better
miniAQLQ Q13: How limited have you been during the past 2 weeks doing moderate activities (such as walking, housework, gardening, shopping, climbing stairs) because of your asthma?	0 (0 to 3.0) *p* = 0.021*	+1.0 (0 to 3.0) *p* = ns	+1.5 (0 to 3.0) *p* = 0.007**	1–7 higher = better
miniAQLQ Q14: How limited have you been during the past 2 weeks doing social activities (such as talking, playing with pets/children, visiting friends/relatives) because of your asthma?	0 (0 to 2.0) *p* = ns	0 (0 to 1.0) *p* = 0.020*	+1.5 (0 to 2.0) *p* = 0.013*	1–7 higher = better
miniAQLQ Q15: How limited have you been during the past 2 weeks doing work-related activities (tasks you have to do at work) because of your asthma?	+1.0 (0 to 1.0) *p* = ns	+1.0 (1.0 to 3.0) *p* = 0.004**	+1.0 (0 to 3.0) *p* = 0.018*	1–7 higher = better
ACQ-6 Total score (MCID ≤ 0.5)	−1.0 (−1.83 to −0.16) *p* = 0.003**	−1.34 (−2.33 to −0.66) *p* = 0.001**	−1.83 (−2.67 to −0.83) *p* = <0.001***	0–6 MCID ≤0.5
ACT Total score (MCID ≥ 3, ≥ 20 = controlled)	+3.5 (0 to 6.0) *p* = 0.003**	+4.0 (3.0 to 10.0) *p* = <0.001***	+7.0 (5.0 to 10.0) *p* = 0.003**	5–25 MCID ≥3
miniAQLQ Total score (MCID ≥0.5)	+0.40 (−0.24 to 1.10) *p* = 0.097	+0.60 (0.27 to 1.67) *p* = 0.005**	+1.30 (0.31 to 2.13) *p* = 0.007**	1–7 MCID ≥0.5

Values are median (IQR). *Δ* denotes change from baseline (follow-up minus baseline). *Δ* 3M/6M/12M = change at 3, 6, 12 months.

MCID sources: ACQ-6 (11), ACT (12), miniAQLQ (13).

ACQ-6, 6-item asthma control questionnaire; ACT, asthma control test; miniAQLQ, mini-Asthma Quality of Life Questionnaire; BSL, baseline; IQR, interquartile range; MCID, minimal clinically important difference.

* *p* < 0.05.

** *p* < 0.01.

*** *p* < 0.001; ns, not significant.

**Table 4:** Wilcoxon signed-rank test results comparing each timepoint with baseline.

Outcome	Comparison	Median difference (Q1, Q3)	Number of comparisons	*p*-value
ACQ-6 (total)				
	M3 vs BSL	−1 (−1.83, −0.16)	21	0.0025**
	M6 vs BSL	−1.34 (−2.33, −0.66)	17	0.0012**
	M12 vs BSL	−1.83 (−2.67, −0.83)	13	0.0002***
ACQ-6 (question 3)				
	M3 vs BSL	−1 (−2, 0)	19	0.0360*
	M6 vs BSL	−2 (−3, 0)	16	0.0045**
	M12 vs BSL	−2.5 (−3, −0.75)	12	0.0084**
ACQ-6 (question 6)				
	M3 vs BSL	−1 (−1, 0)	18	0.0092**
	M6 vs BSL	−1 (−2.25, −1)	16	0.0023**
	M12 vs BSL	−1 (−2, −0.75)	12	0.0079**
ACT (total)				
	M3 vs BSL	3.5 (0.25, 6)	22	0.0034**
	M6 vs BSL	4 (3, 9.25)	18	0.0003***
	M12 vs BSL	7 (5, 10)	13	0.0025**
ACT (question 1)				
	M3 vs BSL	0.5 (0, 1)	18	0.0148*
	M6 vs BSL	1.5 (0.25, 2)	14	0.0047**
	M12 vs BSL	2 (0.25, 2.75)	10	0.0235*
ACT (question 4)				
	M3 vs BSL	0 (0, 1)	18	0.1735
	M6 vs BSL	1 (0, 2)	14	0.0080**
	M12 vs BSL	1 (0.25, 1.75)	10	0.0199*
miniAQLQ (total)				
	M3 vs BSL	0.4 (−0.19, 1.08)	20	0.0966
	M6 vs BSL	0.6 (0.27, 1.6)	16	0.0052**
	M12 vs BSL	1.3 (0.39, 2.06)	12	0.0068**
miniAQLQ (question 12)				
	M3 vs BSL	0 (0, 2)	21	0.0261*
	M6 vs BSL	0 (−1, 2.5)	15	0.1761
	M12 vs BSL	2 (0.25, 2.75)	14	0.0063**
miniAQLQ (question 13)				
	M3 vs BSL	0 (0, 3)	21	0.0213*
	M6 vs BSL	1 (0, 2)	15	0.0934
	M12 vs BSL	1.5 (0.25, 2.75)	14	0.0071**
miniAQLQ (question 14)				
	M3 vs BSL	0 (0, 2)	21	0.2418
	M6 vs BSL	0 (0, 1)	15	0.0201*
	M12 vs BSL	1.5 (0, 2)	14	0.0131*
miniAQLQ (question 15)				
	M3 vs BSL	1 (0, 1)	21	0.0982
	M6 vs BSL	1 (1, 2.5)	15	0.0035**
	M12 vs BSL	1 (0.25, 2.75)	14	0.0176*

*p*-values were obtained from paired Wilcoxon signed-rank tests comparing each follow-up timepoint (M3, M6, M12) with baseline (BSL). Values are presented as median difference (Q1, Q3).

* *p* < 0.05.

** *p* < 0.01.

*** *p* *<* *0.001.*

In the miniAQLQ, all four activity-related items (Q12–15) improved over the 12-month follow-up, with the most consistent and significant changes observed at month 12 (Tables 2–4). Item Q12 (strenuous activities) improved from a median of 2.0 at baseline to 4.0 at month 12 (median change +2.0, *p* = 0.006), with early significant improvement also at month 3 (*p* = 0.026). Item Q13 (moderate activities) showed significant improvement at months 3 and 12 (*p* = 0.021 and *p* = 0.007, respectively). Items Q14 (social activities) and Q15 (work-related activities) reached statistical significance from month 6 onwards (Q14: month 6 *p* = 0.020, month 12 *p* = 0.013; Q15: month 6 *p* = 0.004, month 12 *p* = 0.018). Across all exertional items, the magnitude of improvement was progressive over time, with the largest changes consistently observed at the 12-month timepoint. The temporal pattern of improvement — emerging early for more severe activity limitations and consolidating progressively across all domains — was consistent across the three questionnaire instruments and is illustrated in Figure 1.

## 4 Discussion

The main finding of this study is a progressive and sustained reduction in exertional symptom burden, as captured by exercise-related items of the ACQ-6, ACT and miniAQLQ, over 12 months of tezepelumab treatment in a real-world severe asthma cohort. Changes in these exercise-specific items were at least as pronounced as changes in total questionnaire scores, suggesting that exertional symptoms and activity limitation form a particularly responsive domain under TSLP blockade. This extends the established clinical profile of tezepelumab beyond exacerbation reduction, lung function and type 2 biomarkers, and points to a clinically relevant impact on how patients experience everyday physical activity (26, 27).

One major limitation of this study is the limited sample size. For the ACQ-6 and miniAQLQ total scores, 34 paired observations would be required to detect a mean difference of 0.5 points, assuming a standard deviation of the paired differences of 1.0, with 80% power at a two-sided alpha level of 0.05. For the ACT total score, 16 paired observations would be required to detect a mean difference of 3 points, assuming a standard deviation of the paired differences of 4.0, with 80% power at a two-sided alpha level of 0.05. Although the available sample size was sufficient for the assumed ACT difference; it was below the estimated requirement for ACQ-6 and miniAQLQ; therefore, the overall findings should be interpreted as exploratory. In addition, the number ofstatistical comparisons increase the risk of Type I error, and the reported *p*-values should be interpreted asdescriptive and hypothesis-generating. However, when applying a conservative Bonferroni correction for 33 comparisons, corresponding to a significance threshold of *p* < 0.0015, the changes in ACQ-6 from baseline to month 6 and month 12, as well as the change in ACT from baseline to month 6, remained statistically significant. A limitation of this analysis is the reduced sample size at later follow-up time points (*n* = 20 at 6 months, *n* = 16 at 12 months) due to staggered enrollment and ongoing data collection. Although this does not constitute survivorship bias—as data are missing because patients had not yet reached these time points rather than due to selective dropout—the smaller cohort at 12 months reduces statistical power for detecting longitudinal effects and warrants cautious interpretation of long-term trends.

TSLP blockade modulates the epithelial alarmin cascade upstream of multiple effector pathways. TSLP, and to varying degrees the related epithelial alarmins IL-33 and IL-25, are released from airway epithelial cells following exposure to allergens, respiratory viruses and air pollutants, particularly when the epithelium is stressed or injured, and together they orchestrate type-2-driven inflammation via activation of dendritic cells, group 2 innate lymphoid cells, mast cells and eosinophils, leading to downstream IL-4, IL-5 and IL-13 production, eosinophilic airway inflammation, mucus hypersecretion and structural changes that lower the threshold for exertion-related symptoms (28, 29).

Extrapolating cautiously, one may speculate that gradual anti-inflammatory and anti-remodelling effects of TSLP blockade, accruing over months, could contribute to a higher threshold for exertional symptoms; however, given that only patient-reported outcomes were collected, and no serial lung function or bronchodilation testing was performed, this remains a hypothesis rather than a demonstrated mechanism. Furthermore, whether patients with available 12-month data are fully representative of the original cohort in terms of baseline characteristics cannot be excluded as a potential source of bias, and findings should be interpreted with this caveat in mind.

Improvements exceeding the MCID were observed for ACT and ACQ-6 as early as Month 3, and for miniAQLQ by Month 6, demonstrating the progressive and clinically meaningful effectiveness of tezepelumab — particularly with respect to exertion related symptoms - which is of considerable clinical relevance.

Several limitations should be considered when interpreting these findings. The observational, uncontrolled design precludes causal inference; the observed improvements in exercise-related PRO items may reflect general improvements in asthma control, changes in activity patterns, or regression to the mean rather than a specific effect on exertional airway physiology. We did not collect objective measures of exercise capacity or exertional mechanics (such as the 6-minute walk test, cardiopulmonary exercise testing, or indirect bronchoprovocation), so we cannot determine whether the PRO changes reflect improved physical performance, reduced exertional bronchoconstriction, altered dyspnoea perception, or a combination of these factors. The exercise-related items of the ACQ-6, ACT and miniAQLQ were not developed to capture distinct physiological mechanisms and should therefore be interpreted primarily as indicators of symptom and activity burden during everyday exertion; however, these validated PRO instruments are widely used, responsive to change and provide clinically meaningful, patient-centered information on perceived limitations in daily activities, thereby complementing mechanistic data from controlled trials rather than serving as direct surrogates of specific physiological pathways. Available tezepelumab trials, including CASCADE, have focused on airway inflammation, remodelling and hyperresponsiveness and have not incorporated exertion-specific endpoints such as exercise capacity or dynamic measures of breathing, so the link between reduced airway hyperresponsiveness (AHR) and improved exercise tolerance remains inferential. Finally, this was a single-center, routine-practice cohort without a comparator arm, which limits generalisability and prevents benchmarking against other biologics.

Despite these caveats, our data provide first systematic, longitudinal evidence from routine care that tezepelumab is associated with clinically meaningful improvements in exertion-related symptoms and activity limitation in severe asthma, with benefits emerging and consolidating over 12 months. The observed improvements in exertional symptoms are consistent with – and may reflect – the mechanistic framework in which TSLP blockade attenuates airway hyperresponsiveness, eosinophilic inflammation, and structural remodelling (15).

However, the interpretation of these findings is limited by the small sample size (*n* = 24) and the retrospective observational design, which preclude causal inference and mechanistic conclusions. Future prospective trials with a bigger patient number that combines standardised exercise testing, dynamic measures such as inspiratory capacity during the 6MWT, and serial biomarkers with exertion-focused PROs will be essential to clarify the mechanisms underlying this signal and identify patients most likely to benefit from this therapy.

The clinical relevance of these questionnaire-based improvements is further supported by evidence that ACQ-7 scores independently predict physically limiting breathlessness after adjustment for BMI and exercise capacity, suggesting that the observed changes reflect genuine functional gains rather than subjective perception alone (4).

## Data Availability

The raw data supporting the conclusions of this article will be made available by the authors, without undue reservation.
