# Supplementary material for: Tezepelumab attenuates exertional symptom burden in severe asthma: insights from a real-world cohort
Source: Front Allergy. 2026 Jun 29;7:1849268. doi: 10.3389/falgy.2026.1849268 (PMC13357412; doi:10.3389/falgy.2026.1849268)
Supplement: Supplementary file 1 [file Datasheet1.docx]

Supplementary Material

**Supplementary Table 1. ACQ-6, ACT and miniAQLQ items, with items used in this study highlighted**

| **Instrument** | **Item** | **Item text** | **Response scale** | **Domain** |
| --- | --- | --- | --- | --- |
| ***Asthma Control Questionnaire — 6 items (ACQ-6) \| Past week \| 0–6, higher = worse control*** | | | | |
| **ACQ-6** | Q1 | How often were you woken by your asthma during the night? | *0 (never) – 6 (unable to sleep)* | Nocturnal symptoms |
|  | Q2 | How bad were your asthma symptoms when you woke up in the morning? | *0 (no symptoms) – 6 (very severe)* | Morning symptoms |
|  | **Q3** | How limited were you in your activities because of your asthma? **★** | *0 (not limited) – 6 (totally limited)* | **Activity limitation** |
|  | Q4 | How much shortness of breath did you experience because of your asthma? | *0 (none) – 6 (a great deal)* | Dyspnoea |
|  | Q5 | How much of the time did you have wheezing? | *0 (none) – 6 (all of the time)* | Wheeze |
|  | **Q6** | How many puffs of short-acting bronchodilator (e.g. salbutamol) have you used each day? **★** | *0 (none) – 6 (>16 puffs/day)* | **Reliever use** |
| ***Asthma Control Test (ACT) \| Past 4 weeks \| 5–25, higher = better control*** | | | | |
| **ACT** | **Q1** | How much of the time has your asthma kept you from getting as much done at work, school or home? **★** | *1 (all the time) – 5 (none of the time)* | **Activity impairment** |
|  | Q2 | How often have you had shortness of breath? | *1 (>once/day) – 5 (not at all)* | Dyspnoea frequency |
|  | Q3 | How often did your asthma symptoms wake you up at night or earlier than usual in the morning? | *1 (≥4 nights/week) – 5 (not at all)* | Nocturnal/early symptoms |
|  | **Q4** | How often have you used your rescue inhaler or nebuliser medication (such as salbutamol)? **★** | *1 (≥3 times/day) – 5 (not at all)* | **Reliever use** |
|  | Q5 | How would you rate your asthma control during the last 4 weeks? | *1 (not controlled) – 5 (completely controlled)* | Overall control |
| ***Mini Asthma Quality of Life Questionnaire (miniAQLQ) \| Past 2 weeks \| 1–7, higher = better QoL*** | | | | |
| **miniAQLQ** | Q1 | How often did you feel short of breath as a result of your asthma? | *1 (all the time) – 7 (none of the time)* | Symptoms |
|  | Q2 | How often did you feel bothered by or have to avoid dust in the environment? | *1 (all the time) – 7 (none of the time)* | Environmental stimuli |
|  | Q3 | How often did you experience chest tightness or chest heaviness? | *1 (all the time) – 7 (none of the time)* | Symptoms |
|  | Q4 | How often did you feel frustrated as a result of your asthma? | *1 (all the time) – 7 (none of the time)* | Emotional function |
|  | Q5 | How often were you bothered by or did you have to avoid cigarette smoke in the environment? | *1 (all the time) – 7 (none of the time)* | Environmental stimuli |
|  | Q6 | How often did you wheeze as a result of your asthma? | *1 (all the time) – 7 (none of the time)* | Symptoms |
|  | Q7 | How often did you feel concerned about having asthma? | *1 (all the time) – 7 (none of the time)* | Emotional function |
|  | Q8 | How often did you experience chest tightness when you woke up in the morning? | *1 (all the time) – 7 (none of the time)* | Symptoms |
|  | Q9 | How often were you bothered by or did you have to avoid going outside because of weather or air pollution? | *1 (all the time) – 7 (none of the time)* | Environmental stimuli |
|  | Q10 | How often did you feel afraid of not having your asthma medication available? | *1 (all the time) – 7 (none of the time)* | Emotional function |
|  | Q11 | How often did you cough as a result of your asthma? | *1 (all the time) – 7 (none of the time)* | Symptoms |
|  | **Q12** | How limited have you been doing strenuous activities such as hurrying, exercising, running up stairs, or sports? **★** | *1 (totally limited) – 7 (not limited at all)* | **Activity — strenuous** |
|  | **Q13** | How limited have you been doing moderate activities such as walking, housework, gardening, or shopping? **★** | *1 (totally limited) – 7 (not limited at all)* | **Activity — moderate** |
|  | **Q14** | How limited have you been doing social activities such as talking, playing with pets/children, or visiting friends/relatives? **★** | *1 (totally limited) – 7 (not limited at all)* | **Activity — social** |
|  | **Q15** | How limited have you been doing work-related activities? **★** | *1 (totally limited) – 7 (not limited at all)* | **Activity — work-related** |
