# Supplementary figures and images for: Tezepelumab attenuates exertional symptom burden in severe asthma: insights from a real-world cohort
Source: Front Allergy. 2026 Jun 29;7:1849268. doi: 10.3389/falgy.2026.1849268 (PMC13357412; doi:10.3389/falgy.2026.1849268)

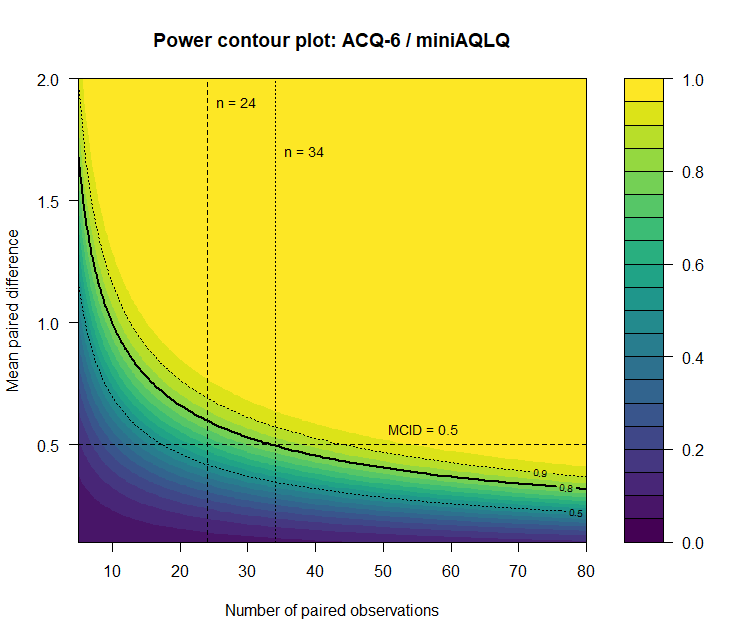

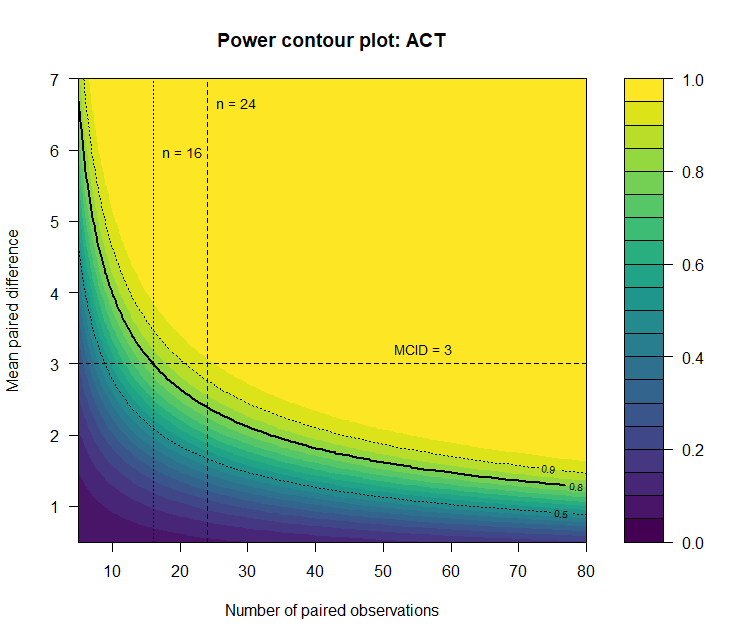

Supplement: Supplementary file 2 [file Supplementaryfile1.docx]
